# Strategies to improve the therapeutic efficacy of mesenchymal stem cell‐derived extracellular vesicle (MSC-EV): a promising cell-free therapy for liver disease

**DOI:** 10.3389/fbioe.2023.1322514

**Published:** 2023-12-13

**Authors:** Lijuan Zheng, Hui Gong, Jing Zhang, Linna Guo, Zhuofan Zhai, Shuang Xia, Zhiyu Hu, Jing Chang, Yizhu Jiang, Xinran Huang, Jingyi Ge, Bikui Zhang, Miao Yan

**Affiliations:** ^1^ Department of Pharmacy, The Second Xiangya Hospital, Central South University, Changsha, China; ^2^ Institute of Clinical Pharmacy, Central South University, Changsha, China; ^3^ International Research Center for Precision Medicine, Transformative Technology and Software Services, Changsha, China; ^4^ Xiangya School of Pharmaceutical Sciences, Central South University, Changsha, China; ^5^ Xiangya School of Medicine, Central South University, Changsha, China

**Keywords:** mesenchymal stem cell, extracellular vesicle, liver disease, therapeutic efficacy, bioengineering

## Abstract

Liver disease has emerged as a significant worldwide health challenge due to its diverse causative factors and therapeutic complexities. The majority of liver diseases ultimately progress to end-stage liver disease and liver transplantation remains the only effective therapy with the limitations of donor organ shortage, lifelong immunosuppressants and expensive treatment costs. Numerous pre-clinical studies have revealed that extracellular vesicles released by mesenchymal stem cells (MSC-EV) exhibited considerable potential in treating liver diseases. Although natural MSC-EV has many potential advantages, some characteristics of MSC-EV, such as heterogeneity, uneven therapeutic effect, and rapid clearance *in vivo* constrain its clinical translation. In recent years, researchers have explored plenty of ways to improve the therapeutic efficacy and rotation rate of MSC-EV in the treatment of liver disease. In this review, we summarized current strategies to enhance the therapeutic potency of MSC-EV, mainly including optimization culture conditions in MSC or modifications of MSC-EV, aiming to facilitate the development and clinical application of MSC-EV in treating liver disease.

## 1 Introduction

Liver disease has emerged as a significant worldwide health challenge due to its diverse causative factors and therapeutic complexities. Most liver diseases eventually progress to end-stage liver disease but lack effective treatments. Liver transplantation remains the only effective therapy with accompanying limitations of donor organ shortage, lifelong immunosuppressants and expensive treatment cost (Hu et al., 2023). As an emerging therapeutic approach, stem cell therapy has shown promising prospects in multiple liver diseases (Yang et al., 2021). Mesenchymal stem cells (MSCs) are mesoderm-derived pluripotent stem cells that can be extracted from a wide range of tissues, including bone marrow, adipose, and umbilical cord. Liver stem cells are also present in liver tissue, which also is the potential source of MSCs (Bruno et al., 2021). MSCs exhibit self-renewal, multiline differentiation and immunomodulatory properties, and have the potential to repair or regenerate damaged tissues and inhibit immune response (Matsuzaka and Yashiro, 2022).

In recent years, more and more studies have shown that the therapeutic effect of MSCs is mainly dependent on their paracrine function (Meirelles et al., 2009). Due to concerns regarding the low cell survival, undesired differentiation, tumorigenicity, emboli formation and ethical issues associated with transplanting cells directly, there have been proposals to utilize cell-based “cell-free therapy,” which refers to utilizing MSC secretomes to replace the cells for treatment. Extracellular vesicle (EV) is the main paracrine secretion of MSCs, playing a crucial role in intercellular communication. MSC-EV can be isolated and purified from the supernatant of the culture medium (Watanabe et al., 2021). According to size and release mode, EVs can be further classified into three major categories: exosomes (30∼100 nm), microvesicles (100∼1000 nm), and apoptotic vesicles (500∼2000 nm) (Hu et al., 2020). MSC-EV carries genetic material and bioactive substances (chemokines, miRNAs, DNA, proteins, and lipids) from the parental cells, thus retaining the therapeutic properties of MSC (Baek et al., 2019; Harrell et al., 2019).

Numerous preclinical studies have confirmed the efficacy of MSC-EV in liver disease (Ding et al., 2022). Several studies have suggested that MSC-EV may exhibit superior biological efficacy compared to MSCs in treating liver disease, which may be attributed to the enrichment of therapeutic factors from the parental cells in MSC-EV, as well as their specific affinity to liver (Tamura et al., 2016; Yao et al., 2019; Rostom et al., 2020; El-Derany and AbdelHamid, 2021). Despite the many potential advantages of natural MSC-EV, many inherent limitations constrain its clinical translation, like the heterogeneity, low yield and rapid elimination *in vivo*, which may affect their therapeutic efficacy and quality. Therefore, scientists are designing engineered MSC-EV to improve their therapeutic efficacy and rotation rate *in vivo*.

To the best of our knowledge, this is the first report to summarize the engineering strategies to improve the efficacy of MSC-EV in liver disease. In this review, we summarize current strategies for enhancing the therapeutic potency of MSC-EV, including optimization culture conditions in MSC or modifications of MSC-EV, aiming to facilitate the development and clinical application of MSC-EV in treating liver disease.

## 2 Application of MSC-EV in liver disease

Liver disease is characterized by inflammation, tissue damage, and impaired liver function (Vasques et al., 2022). A substantial number of preclinical studies have confirmed the efficacy of MSC-EV in liver diseases such as acute liver injury (ALI)/hepatic failure (AHF), hepatic fibrosis (AHF)/cirrhosis, hepatic ischemia-reperfusion injury (HIRI), non-alcoholic steatohepatitis (NASH), and autoimmune liver diseases (AIH), which were summarized in Table 1. Currently, MSC-EV used for liver disease therapeutic research are mainly derived from umbilical cord, bone marrow and adipose. In addition, some other sources of MSC, such as liver stem cells (LSCs), also share the common characteristics of MSC. LSC-EV can also be used for the treatment of liver diseases, such as promoting liver regeneration in hepatectomy models and anti-fibrosis. In summary, according to current studies, MSC-EV mainly alleviates liver injury through immunomodulation, regulation of tissue cell proliferation, anti-fibrosis, and its ability to stimulate angiogenesis and anti-oxidative stress.

**TABLE 1:** The application and mechanism of MSC-EV in liver diseases.

Cell source	Type of EV	EV cargo	Liver disease	Experimental model	Mechanism of action	Ref.
rat bone marrow MSC	exosome	CD44 and OTUB1	acute liver injury (ALI)	*in vivo*: CCl_4_-mice	• suppressed hepatocyte’s ferroptosis by maintaining SLC7A11 function	Lin et al. (2022a)
				*in vitro*: CCl_4_-primary hepatocytes		
human umbilical cords MSC	exosome	—	acute liver injury (ALI)	*in vitro*: LPS-RAW246.7 cells *in vivo*: LPS/D-gal-mice	• inhibited activation of the NLRP3 inflammasome both *in vitro* and *in vivo*	Jiang et al. (2019)
human umbilical cords MSC	exosome		acute liver injury (ALI), liver fibrosis	*in vitro*: CCl_4_-L02 cells	• decreased oxidative stress and apoptosis	Jiang et al. (2018)
				*in vivo*: CCl_4_-mice		
bone marrow MSCs	exosome	—	acute liver failure (ALF)	*in vitro*: D-gal/LPS-primary hepatocytes	• attenuated hepatocyte apoptosis by promoting autophagy	Zhao et al. (2019)
human adipose MSC	EV	lncRNA H19	acute liver failure (ALF)	*in vivo*: LPS/D-gal-rat *in vitro*: LPS/D-gal-primary hepatocytes	• promoted hepatocyte regeneration and protecting apoptotic hepatocytes via the HGF/c-Met pathway	Jin et al. (2018)
					• increased the survival rate of rats with ALF	
human umbilical cords MSC	exosome	—	acute liver failure (ALF)	*in vivo*: APAP-mice	• inhibited oxidative stress-induced apoptosis via upregulation of ERK1/2 and PI3K/AKT signaling pathways.	Wu et al. (2021)
				*in vitro*: APAP-L02 cells		
adipose MSC	exosome	miR-17	acute liver failure (ALF)	*in vitro*: LPS-RAW 246.7 cells *in vivo*: LPS/D-gal-mice	• suppressed NLRP3 inflammasome activation by targeting TXNIP	Liu et al. (2018)
					• reduced inflammatory factor secretion	
human bone marrow MSC	exosome	let-7a-5p	acute-on-chronic	*in vivo*: CCl_4_-mice	• targeted MAP4K3 to reduce TFEB phosphorylation	Lin et al. (2022b)
			liver failure (ACLF)	*in vitro*: CCl_4_-L02 cells	• Promoted autophagy-lysosomal fusion at the endstage of autophagic flux	
placenta mesenchymal stem cells (PD-MSCs)	exosome	CRP	hepatic failure	*in vivo*: BDL-rats	• upregulated Wnt signaling pathway and angiogenesis	Jun et al. (2020)
				*in vitro*: LCA-HUVECs and WB-F344 cells		
rat adipose MSC	exosome	—	hepatic ischemia-reperfusion injury (HIRI)	*in vivo*: block the blood vessel of liver-rats	• inhibited the NF-κB pathway and activate the Wnt/β-catenin pathway	Piao et al. (2022)
umbilical cords MSC	EV	CCT2	hepatic ischemia-reperfusion injury (HIRI)	*in vivo*: block the blood vessel of liver -mice	• regulated the calcium channels to affect Ca2+ influx and suppress CD154 synthesis in CD4+T cells	Zheng et al. (2020)
				*in vitro*: PMA^+^ionomycin - CD^4+^ T cells		
human umbilical cords MSC	EV	MnSOD	hepatic ischemia-reperfusion injury (HIRI)	*in vitro*: H_2_O_2_-L02, LPS-neutrophils	• protected against hepatic apoptosis by reducing the infiltration of neutrophils and alleviating oxidative stress in hepatic tissue	Yao et al. (2019)
				*in vivo*: block the blood vessel of liver-mice		
human umbilical cords MSC	exosome	miR-1246	hepatic ischemia-reperfusion injury (HIRI)	*in vitro*: Hypoxia-reoxygenation (H/R) -L02 cells	• presented anti-apoptotic and pro-survival effects by regulating GSK3β-Wnt/β-catenin pathway.	Xie et al. (2019a)
				*in vivo*: block the blood vessel of liver -mice		
human umbilical cords MSC	exosome	miR-1246	hepatic ischemia-reperfusion injury (HIRI)	*in vitro*: Hypoxia-reoxygenation (H/R)-L02 cells	• decreased Th17/Treg ratio in CD4^+^ T cells via the IL-6/gp130/STAT3 axis	Xie et al. (2019b)
				*in vivo*: block the blood vessel of liver-mice		
human-induced pluripotent stem cell–derived MSC	exosome	—	hepatic ischemia-reperfusion injury (HIRI)	*in vivo*: block the blood vessel of liver -mice	• presented hepatoprotective and proliferative effects by regulating S1P-SK1 pathway	Du et al. (2017a)
mice bone marrow MSC	exosome	miR-223-3p	autoimmune hepatitis (AIH)	*in vivo*: S100/CFA-mice	• regulated the expression of IL-1β and IL-6 in the liver	Lu et al. (2019)
				*in vitro*: RAW264.7 cells	• altered the proportions of Treg and Th17 cells in the spleen of mice	
human umbilical cords MSC	exosome	—	non-alcoholic steatohepatitis (NASH)	*in vivo*: methionine–choline-defcient (MCD) diet-mice	• regulated the anti-inflammatory phenotype of macrophages	Shi et al. (2022)
					• reversed PPARα protein expression in liver cells	
human adipose MSC	Small EV	—	non-alcoholic steatohepatitis (NASH) with rapid accumulation of fibrosis	*in vivo*: LPS-mice	• Increased anti-inflammatory macrophages in the liver	Watanabe et al. (2020)
					• Alleviated liver fibrosis but not affect fat accumulation	
rat bone marrow MSC	exosome	—	non-alcoholic steatohepatitis (NASH)	*in vivo*: high fat diet (HFD)-rats	• prevented hyperlipidemia	El-Derany and AbdelHamid (2021)
					• reduced hepatic steatosis, liver apoptosis and mitochondrial mitophagy	
amnion MSC	EV	—	non-alcoholic steatohepatitis (NASH), liver fibrosis	NASH model: HFD-rats liver fibrosis model: CCl4-rats	• decreased the mRNA expression levels of inflammatory cytokines	Ohara et al. (2018)
					• decreased flber accumulation, Kupffer cells (KCs) number, and hepatic stellate cell (HSC) activation in rats	
					• suppressed the LPS/TLR4 signaling pathway	
mice bone marrow MSC	Small EV	IL-10	Traumatic hemorrhagic shock (THS) induced hepatic injury	*in vitro*: LPS-RAW264.7 cells	• facilitated PTPN22 levels	Zhang et al. (2021)
				*in vivo*: Fixed volume shock surgery-mice	• promoted Kupffer cell polarization	
human placental MSC	EV	circ-RBM23	partial hepatectomy (PH)	*in vivo*: 70% partial hepatectomy-mice	• regulated liver regeneration via the miR-139-5p/RRM2/AKT/mTOR pathway	Li et al. (2022)
				*in vivo*: AML-12 and L02 cells		
human umbilical cords MSC	exosome	miR-124	partial hepatectomy (PH)	*in vivo*: 70% partial hepatectomy-rats	• promoted rat liver cell proliferation via suppressing Foxg1 expression	Song et al. (2021)
				*in vitro*: BRL-3A Rat liver cells		
human umbilical cords MSC	EV	—	schistosomiasis	*in vivo*: S.japonicum-infected mice	• suppressed hepatic stellate cell proliferation and activation	Dong et al. (2020)
				*in vitro*: TGF-β1 -LX_2_ cells		
human adipose MSC	exosome	—	liver fibrosis	*in vivo*: DEN/CCl4-mice	• suppressed HSCs activation	Wu et al. (2022)
				*in vitro*: TGF-β-LX_2_ cells/AML12 cells/mice HSCs	• remodeled glutamine and ammonia metabolism mediated by hepatocellular glutamine synthetase	
human umbilical cords MSC	exosome	miR-148a	liver fibrosis	*in vivo*: CCl_4_-mice *in vitro*: LPS/IFN-γ/IL-4-RAW264.7 cells	• regulated intrahepatic macrophage functions through KLF6/STAT3 signaling	Tian et al. (2022)
human umbilical cords MSC	exosome	BECN1	liver fibrosis	*in vivo*: CCl_4_-mice	• induce HSC ferroptosis via the downregulation xCT/GPX4 pathway	Tan et al. (2022)
				*in vitro*: LX_2_ cells		
human bone marrow MSC	exosome	CIRCD01	liver fibrosis	*in vitro*: LX_2_ cells	• suppressed HSC activation by miR-141-3p/PTEN/AKT pathway	Ma et al. (2022a)
human bone marrow MSC	exosome	circCDK13	liver fibrosis	*in vivo*: TAA-mice	• inhibited PI3K/AKT and NF-κB signaling pathways by regulating the miR-17-5p/KAT2B axis	Ma et al. (2022b)
human Wharton jelly MSC	exosome	—	liver fibrosis	*in vitro*: TGF-β-LX_2_ cells	• inhibited NOXs pathway and phosphorylation of Smad3C protein	Afarin et al. (2022)
bone marrow MSC	exosome	miR-618	liver fibrosis	*in vivo*: CCl_4_-mice	• attenuated the progression of liver fibrosis via targeting Smad4	Sun et al. (2022)
				*in vitro*: TGF-β-LX_2_ cells		
human tonsil MSC	Small EV	miR-486-5p	liver fibrosis	*in vivo*: CCl_4_-mice	• inactivated HSCs by suppressing hedgehog signaling	Kim et al. (2021a)
				*in vitro*: primary hepatic stellate cells		
mice adipose MSC	EV	miR-150-5p	liver fibrosis	*in vivo*: CCl_4_-mice	• inhibited HSC proliferation by inhibiting the CXCL1 expression	Du et al. (2021)
				*in vitro*: TGF-β-primary HSCs		
rat bone marrow MSC	EV	—	liver fibrosis	*in vivo*: CCl_4_-rats	• reversed hepatocellular damage	Rostom et al. (2020)
					• ameliorate hepatic fibrosis and regressed collagen deposition in the liver tissue	
human bone marrow MSC	exosome	—	liver fibrosis	*in vivo*: CCl_4_-rats	• Inhibited HSC activation through the Wnt/β-catenin pathway	Rong et al. (2019)
				*in vitro*: HSC cells		
human Tonsil MSC	CM	IL-1Ra	liver fibrosis	*in vivo*: CCl_4_-mice	• reduced inflammation and fibrosis	Kim et al. (2019a)
human liver stem cell	EV	—	non-alcoholic steatohepatitis (NASH), liver fibrosis	*in vivo*: methionine–choline-deficient (MCD) diet-mice	• improve liver morphology, ameliorating fibrosis and inflammation by downregulating fibrosis-associated genes	Bruno et al. (2020)
human embryonic stem cells-derived MSC	EV	—	cirrhosis	*in vivo*: TAA-rats	• suppressed the proliferation of peripheral blood mononuclear cells	Mardpour et al. (2018)
					• increased the secretion of anti-inflammatory cytokines (TGF-β and IL-10) and decreased IFN-γ	
					• reduced fibrosis and collagen density, necrosis, caspase density, portal vein diameter and transaminitis	
rat bone marrow MSC	exosome	—	cirrhosis	*in vivo*: CCl_4_-rats	• restrained hepatocyte pyroptosis	Zhang et al. (2022)
				*in vitro*: CCl_4_-BRL rat hepatocytes		

Existing reports suggest that MSC-EV has a potent immunomodulatory effect on immune cells, inflammatory vesicles, and the release of inflammatory factors, thereby modulating the immune microenvironment within injured tissues. For example, in the mouse model with hepatic injury or liver disease, MSC-EV can modulate the function of kupffer cells and polarize macrophages from M1 (pro-inflammatory phenotype) to M2 (anti-inflammatory phenotype), regulating the intrahepatic inflammatory microenvironment and repairing damage (Zhang et al., 2021; Shi et al., 2022; Tian et al., 2022). MSC-EV also inhibited the activation of caspase-1 and NLRP3 inflammasome, attenuating the inflammatory response and cellular death (Chen et al., 2018a; Liu et al., 2018; Zhang et al., 2020). In addition, it has been shown that MSC-EV also regulated the expression of inflammatory factors such as IL-1β and IL-6 and reduced the ratio of Treg/Th17, thereby attenuating liver disease (Lu et al., 2019).

MSC-EV can also facilitate liver repair by regulating the fate of liver cells. Exosomes derived from adipose-derived mesenchymal stem cells (ADSCs-Exo) could effectively inhibit the expression of pyroptosis-related factors (such as NLRP3, ASC, caspase-1, and GSDMD-N) and promote the expression of those factors related to liver regeneration (such as Cyclin D1 and VEGF) in HIRI rat (Piao et al., 2022). In addition, the researchers also discovered that MSC-EV had a protective role against ferroptosis by maintaining SLC7A11 function, thus proposing a novel therapeutic strategy for ferroptosis-induced ALI (Zhao et al., 2019). MSC-EV also reduces apoptosis by increasing autophagy in hepatocytes. Studies showed that after injecting MSC-EV, the autophagy-related markers such as LC3 and Beclin-1 are increased and have led to autophagosome formation by hepatocytes. Also, the expression level of apoptosis-related proteins such as Bax and cleaved caspase 3 was decreased (Zhao et al., 2019; Yang et al., 2020; Zhang et al., 2020). MSC-EV can also facilitate liver repair by promoting hepatocyte regeneration. Song et al. found that human umbilical cord blood mesenchymal stem cell (hUCB-MSC) derived exosome promote liver regeneration in rats after partial hepatectomy (PH) via downregulating Foxg1 (Song et al., 2021).

For the treatment of liver fibrosis, MSC-EV can inhibit the abnormal activation of hepatic stellate cells (HSC) and reduce collagen accumulation (Ma et al., 2022; Wang et al., 2022). It was shown that in TAA or CCl_4_-induced HF mouse models, MSC-EV could inhibit HSC activation and reduce collagen accumulation in the liver (Du et al., 2021; Ma et al., 2022). Furthermore, Rong et al. found that the therapeutic effect of MSC-exo against liver fibrosis was significantly greater than that of MSC, based on the measurement of the collagen area, Ishak fibrosis score, MDA levels, IL-1, and IL-6 (Rong et al., 2019). In addition, it has been suggested that mechanisms of pro-angiogenesis (Jun et al., 2020) and anti-oxidative stress (Jiang et al., 2018; Wu et al., 2021) also contribute to the regenerative effects of MSC-EV.

From the available studies, it appears that the therapeutic potential of MSC-EV is not attributable to a single effector, but may work synergistically through multiple substances in the cargo.

## 3 *In vivo* fates of MSC-EV

Understanding the *in vivo* fates of MSC-EV is crucial for optimizing their therapeutic potential, as their biodistribution and retention in target tissues can significantly impact treatment outcomes (Murali and Holmes, 2021; Lui and Leung, 2022). There are many methods for *in vivo* tracer of MSC-EV. Firstly, MSC-EV need to be labeled by lipophilic dyes, membrane-penetrating compounds, or radioactive materials (Qin et al., 2021). Then, the labeled EVs were administrated into the body. Molecular imaging techniques and optical imaging, such as magnetic resonance imaging (MRI), X-ray computed tomography (CT) imaging, magnetic particle imaging (MPI), single-photon emission computed tomography (SPECT), positron emission tomography (PET), fluorescence and bioluminescence imaging, can be employed to visualize the absorption, distribution, metabolism, and excretion of MSC-EV in living organisms (Kim et al., 2019; Arifin et al., 2022). In-depth pharmacokinetic studies of MSC-EV can aid in identifying and optimizing the dosing regimen, thereby ensuring its safety and efficacy.

The biodistribution of MSC-EV is a dynamic process. Unlike MCS tends to get stuck (physical trapping) in the capillary beds of the lungs upon entering the body, MSC-EV can successfully bypass the pulmonary entrapment, circulate in the blood system, and cross the vascular barrier to enter the tissues smoothly (Tamura et al., 2016). This may be due to MSC-EV having a smaller size (Watanabe et al., 2020).

Regardless of the cellular or tissue source, EV injected into the veins of mice is always preferentially distributed in the organs with a mononuclear phagocyte system (MPS) such as the liver, spleen, lungs, and kidneys (Kim et al., 2019). *In situ* analysis showed that the liver was the major organ of small-EVs(<100 nm) localization in the first hour after administration, while distribution to the lungs and spleen peaked between 2 and 12 h. Large-EVs (>200 nm) were most abundant in the lungs in the first hour, followed by a decrease in the lung and an increase in the liver between 2 and 12 h (Wiklander et al., 2015; Morishita et al., 2017; Kang et al., 2021). Such an accumulation in the liver may be determined by the liver’s physiological characteristics and the immune system’s response (Hu et al., 2023). The liver is characterized by large volume, high vascularity, and high metabolism, which means that EV has a higher chance of being absorbed by the liver through blood circulation (Yang et al., 2021). The greater permeability of the hepatic sinusoid facilitates the nanoparticles to deposite in this organ (Zhang et al., 2016; Bruno et al., 2021) MSC-EV is easier to interact with lipophilic tissues because of the lipid bilayer membrane. Coincidently, the liver is a highly lipophilic organ (Mulcahy et al., 2014; Matsuzaka and Yashiro, 2022) The presence of large numbers of kupffer macrophages and lymphocytes in the liver which recognize and uptake MSC-EV. Thus, the liver is an excellent target for EV-based therapy (Borrelli et al., 2018). Interestingly, injury can further increase MSC-EV accumulation in tissues. For example, within the liver failure model, regardless of the route of administration, *in vitro* imaging 6 h after administration showed that liver accumulation of EV in the liver failure mice was higher than that in the normal mice, implying that MSC-EV may have an injurious tissue-targeting property (Haga et al., 2017; Zheng et al., 2020). This injury-targeting property allows MSC-EV to rapidly migrate and localize to the injured liver after systemic injection, which may be due to the activation of kupffer cells at inflammatory sites in the liver and increased uptake of MSC-EV.

EV has a very short half-life *in vivo*. MSC-EV can enter target cells through phagocytosis, macrocytosis, membrane fusion, and receptor-mediated-endocytosis, and then release the bioactive substances it contains and exert therapeutic effects (Gurung et al., 2021). In addition to MSC-EV uptake by target cells, the remaining MSC-EV will be predominantly taken up by immune cells. These immune cells can specifically recognize receptors on the surface of MSC-EV, and then phagocytosis them. Macrophages play a critical role in the elimination of MSC-EV *in vivo*. When depleting macrophages in the organism, the rate of EV clearance decreased and the circulation time in the body increases significantly (Imai et al., 2015; Mats et al., 2020). In addition, many immune cells are also present in the liver and spleen, which also become the main organs and sites for eliminating MSC-EV (Mulcahy et al., 2014). Furthermore, there are reports to reveal that the nanomaterials are not exclusively cleared by immune cells and the contribution of scavenger endothelial cells is also considerable, particularly hepatic sinusoidal endothelial cells (LSECs) (Hayashi et al., 2020). However, whether MSC-EV is currently cleared by other resident cells in the liver needs further investigation.

The surface membrane molecules of MSC-EV are highly relevant to their recognition by liver or immune cells. For example, CD44 on the membrane surface of MSC-EV is one of the molecules involved in recognition in the injured liver. In a mouse model of acute liver injury, the localization of MSC-EV in the injured liver was significantly reduced after the investigators neutralized CD44 expression using antibodies (Lin et al., 2022). In addition, Expression of integrin αvβ5, phosphatidylserine (PS), immunoglobulins, tetraspanins, and lectins also confers a high degree of liver-targeting associated with Kupffer cells (Miyanishi et al., 2007; Hoshino et al., 2015; Murphy et al., 2019). Except for the membrane composition, various factors can affect the absorption, distribution, metabolism, and excretion (ADME) of MSC-EV either directly or indirectly. The choice of dosing regimen, including dosages, frequency, routes, and timing of administration, may also affect the survival time, and therapeutic effects of MSC-EV (Wiklander et al., 2015; Di Rocco et al., 2016). Besides, the size of MSC-EV also affects its elimination *in vivo*. Larger particles can be recognized and cleared by macrophages faster than smaller particles (Kang et al., 2021).

Although some MSC-EV accumulates rapidly in the liver after intravenous injection, a portion can still enter other organs and tissues. Even for MSC-EV that have entered the liver, the vast majority are phagocytosed and cleared by immune cells and liver resident cells. It is important to note that hepatic macrophage recognition and uptake of MSC-EV may be a double-edged sword; on the one hand, MSC-EV may interact with macrophages to inhibit hepatic macrophage activation and regulate hepatic inflammation, and on the other hand, hepatic macrophage recognition may further accelerate the clearance of MSC-EV. Knowing in detail the interrelationship between MSC-EV and hepatic macrophages may further help us better understand the *in vivo* fates of MSC-EV.

Therefore, it is still important to further improve the targeting of MSC-EV to the liver and increase the residence time. Based on the known distribution and elimination characteristics of MSC-EV *in vivo*, it can give us a lot of insights into improving the rotation rate of MSC-EV. An interesting concept exists in the field of nanomedicine research: the stealth effect, where nanoparticles exhibit dose-dependent nonlinear pharmacokinetics because of saturating or depressing bio-clearance of the reticuloendothelial system (RES). Considering that MSC-EV is also a kind of nanoscale vesicle, therefore we can learn from the “stealth effect” of nanomedicines. For example, we can block RES clearance, such as using immunosuppressive drugs to deplete immune cells in the liver before MSC-EV injection., or by modifying the surface properties of MSC-EV to evade the recognition of the immune system, thus achieving long circulation of MSC-EV *in vivo* (Wen et al., 2023).

## 4 Strategies to improve the therapeutic potential of MSC-EV

**FIGURE 1:**
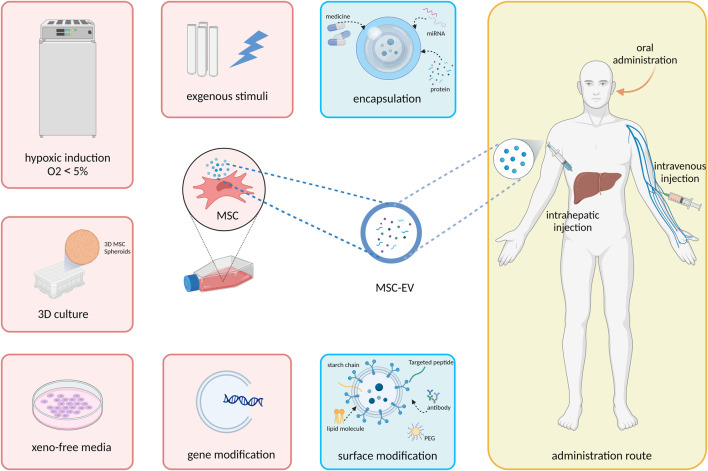
Strategies to improve the therapeutic efficacy of MSC-EV in treating liver disease.

MSC-EV are characterized by their plasticity and can be greatly modified by various extrinsic factors. Thus, *in vitro* preconditioning of MSC-EV is being explored in a variety of ways to enhance their therapeutic potential and *in vivo* fate, including modulation of MSC culture condition, add exogenous cytokines or pharmacological agents, modification of EV cargo and membrane surface proteins, and adjustment of delivery system and route (Figure 1 ). In this section, we will summarize and discuss how these bioengineering techniques can be exploited to improve the efficacy of EVs (Table 1).

### 4.1 Improve the effect and yield of MSC-EV

Even though MSC-EV have a strong potential in regenerative medicine, their low yield under routine culture conditions tends to serve as a major barrier to utilization. Thus, strategies to increase their yield without compromising their functionality are urgently required. The microenvironments in which MSCs are cultivated can alter cells’ proliferation, differentiation, and secretion of bioactive substances, thus influencing the therapeutic potential of MSC-EV (Patel et al., 2018). High-quality MSC-EV has better bioactivity which can promote tissue repair and regeneration, inhibit inflammatory responses and regulate immune functions. Mimicking the tissue-native microenvironment of MSC is one of the strategies for improving the performance of MSC-EV, including providing low oxygen concentration levels and 3D cultures. In addition, adding exogenous stimulatory factors can induce MSCs to secrete anti-tissue damage factors in advance. Furthermore, the main effectors of MSC-EV can be overexpressed by genetic engineering approaches.

#### 4.1.1 Hypoxia induction

Oxygen concentration is essential for the proliferation, differentiation, and self-renewal of MSC. The common oxygen level in standard cell culture is approximately 21% O_2_. However, it has been found that hypoxia-induced MSC showed a significant increase in EV releasing and protein cargo concentrations compared to those in conventional culture conditions (Liu et al., 2020). This may be due to that the low oxygen environment (2∼5% O_2_) is closer to the realistic oxygen levels *in vivo* (Yin et al., 2019). The physiological oxygen concentration in tissues varies from 1% to 12% in peripheral blood. Indeed, MSC is also frequently found in anatomical sites with low oxygen perfusion, such as the bone marrow (4∼7%) and adipose regions (10∼15%) (Madrigal et al., 2014).

Available oxygen tensions for stem cell culture range from normoxic (21% O_2_) to oxygen-deprived conditioned (0.5%–2% O_2_) (Gwam et al., 2021). Oxygen concentration is closely linked to the maintenance of stemness (Mas-Bargues et al., 2019). Hypoxia not only allows the MSC to retain an undifferentiated phenotype but also triggers MSC self-renewal and proliferation (Madrigal et al., 2014; Mas-Bargues et al., 2019; Nazarie Ignat et al., 2021). Furthermore, hypoxia-cultured MSC released more EVs and influenced the expression of EV cargo related to regulatory proteins (Bister et al., 2020; Braga et al., 2022; Jiang et al., 2022).

Numerous studies have shown that EVs derived from hypoxia-conditioned MSC have a higher regenerative capacity than those obtained under normoxia (Pulido-Escribano et al., 2022). The possible mechanism is closely related to hypoxia-induced factors (HIFs) (Jiang et al., 2022). HIF is a transcription factor that mainly regulates cellular adaptation to the hypoxic environment. HIF has direct and indirect pleiotropic effects, modulating the expression of hundreds of molecules involved in processes such as inflammation, migration, proliferation, differentiation, angiogenesis, metabolism, and cell apoptosis (Chang et al., 2013; Yu et al., 2013; Xue et al., 2018; Gao et al., 2021; Ge et al., 2021; Gorgun et al., 2021). Expression of these molecules is reflected in the contents of secreted EV, thus improving the MSC-EV tissue repair ability (Pulido-Escribano et al., 2022).

It was found that the efficacy of hypoxic-induced MSC-EV is indeed increased in liver diseases (Lee et al., 2016; Temnov et al., 2019). EV derived from MSC incubated under 10%O_2_ conditions improved the regenerative process of ALF rats more significantly compared with the control group and the normoxic incubation group (Temnov et al., 2019). Hypoxia treatment enriches miR-182-5p in MSC-EV, enhances immunomodulation and promotes liver regeneration through FOXO1-mediated macrophage polarization (Xu et al., 2022). It has also been found that among the MSC secretion cultured at different pO_2_ (including 21%, 10%, 5% and 1%), the secretion with a pO_2_ of 1% appears to be optimum in cell culture, causing stem cells to release the secretion with the highest liver repair and regeneration capacity (Lee et al., 2017).

Hypoxia preconditioning of MSC with a high potential for clinical use in regenerative medicine. However, the low oxygen environment can increase the cost of culture and management of MSC-EV, and optimal time exposure and degree of hypoxia also need to be further investigated.

#### 4.1.2 3D culture

Traditional 2D adherent monolayer culture systems have many limitations, such as insufficient yield, the need for frequent digestion and subculturing of cells, and a high risk of contamination. More importantly, 2D culture does not adequately mimic the native 3D cellular microenvironment *in vivo*, thus potentially leading to phenotypic changes in MSC and impairments in homing and migration abilities. 3D cell culture and scaffolding technology provide growth conditions closer to the *in vivo* environment and promote the production and function of MSC-EV (Baker and Chen, 2012). 3D culture refers to the culture of cells in a three-dimensional space, so that the cells can migrate and grow in the 3D structure of the carrier, constituting a three-dimensional cell-carrier complex, which allows MSCs to produce more MSC-EV in a shorter time and a smaller volume (Miceli et al., 2019).3D culture has appropriate void structure, mechanical strength, and biocompatibility, providing realistic cell-cell and cell-substrate interactions. Thus, MSCs are better able to maintain their biological activities and functions in a 3D culture environment (Phelps et al., 2018). 3D culture can enrich therapeutically relevant miRNAs and protein cargos in MSC-EV and has stronger trauma repair and regeneration ability, and 3D-MSC-EV is superior to 2D-MSC-EV in promoting the growth of senescent MSCs, decreasing their ROS levels, and maintaining mitochondrial function (Yuan et al., 2022). It has also been shown that 3D culture of MSC enhances its antifibrotic potential and that MSC spheroids reduce tissue fibrosis in a mouse model of liver cirrhosis (Zhang et al., 2016). 3D culture can be categorized into static culture (spheroids, hydrogel scaffolds) and dynamic culture (bioreactors like flat-bottomed flasks or rotating-wall vessels) (McKee and Chaudhry, 2017). Suitable scaffold structures and materials are critical for the success of 3D cultures. The scaffold’s pore structure, surface properties and biocompatibility can influence MSC attachment, growth, and EV release of MSC (Qazi et al., 2017).

#### 4.1.3 Exogenous stimuli

Cellular stress can dramatically alter the internal and external composition of MSC-EV (Borrelli et al., 2018). Studies have shown that MSC therapeutic activity is stimulated by physiological need (Madrigal et al., 2014). Thus, to mimic the microenvironment of the injured tissue, adding exogenous stimulatory substances may be a potential strategy to improve the paracrine efficiency of MSC and induce MSC-EV to contain more potential therapeutic factors.

Adding inflammatory factors into the culture medium is a kind of widely studied preconditioning method to improve liver disease therapeutic effect. The inflammatory factors reported include TNF-α, IL-6, and IFN-γ. TNF-α are considered important signaling molecules released after tissue injury. It was demonstrated that the exosome derived from TNFα pretreated MSC (T-Exo) had better anti-inflammatory effects in the LPS/D-gal-induced ALF mouse model. T-Exo suppressed the expression of NLRP3-related pathways and improved the inflammatory response (Zhang et al., 2020). IL-6 is an important initiator of the uncontrolled cytokine storm. Exosomes derived from IL-6 pretreated MSC improved CCl4-induced tissue damage in ALI mice by inhibiting macrophage activation, reducing pro-inflammatory cytokine expression, and stimulating hepatocyte proliferation (Shao et al., 2020). IFN-γ are often reported to change the properties of MSCs under inflammatory conditions. Another research has shown that EVs derived from IFN-γ pretreated MSC exert higher anti-inflammatory and anti-fibrotic effects on liver fibrosis mice by inducing anti-inflammatory macrophages and regulatory T cells (Takeuch et al., 2021).

In addition to inflammatory factors, other compounds or drugs have also been noted to increase MSC-EV efficacy when used to pretreat MSC. For example, pretreating MSC with NaHS enhances the hepatoprotective effect of MSC-EV on ischemia-reperfusion liver injury in mice. It improves liver function by reducing inflammatory cytokines, apoptosis, liver total oxidative state levels and liver transaminases (Sameri et al., 2022). Traditional Chinese medicine pretreatment is also a novel strategy. It was found that the exosome derived from baicalin-pretreated MSC (Ba-MSC-Exo) significantly attenuated LPS/D-gal-induced acute liver injury and inhibited hepatocyte iron death via the P62/Nrf2/keap1 signaling pathway (Zhao et al., 2022). In addition, co-culturing MSC with hepatocytes in advance could increase the expression of cytokines such as IL-6 and IL-10 in MSC-CM, which had a better therapeutic effect on damaged L02 cells induced by D-gal as well as the ALF rat model (Chen et al., 2018b).

Besides, chemical compounds, such as LPS (Ti et al., 2015), HIF (Gómez-Ferrer et al., 2021), thrombin (Sung et al., 2019), platelet-derived growth factor (PDGF) (Lopatina et al., 2014), NO (Du et al., 2017), and even physical factor such as blue light (Yang et al., 2019), has also been used to promote the EV secretion as well as to enhance the pro-angiogenic, inflammation-regulating capacity of MSC-EV, however not yet be used in liver disease.

Existing studies have shown that appropriate pretreatment of MSC can improve the performance of MSC-EV. When pretreating MSC-EV, the molecular compounds stimulator should be compatible with FDA standards so that EVs produced by these stimulated cells can be used in the clinic. Excessive stimulation may lead to MSC damage or apoptosis, thus affecting the yield and quality of MSC-EV. Therefore, when used in the production of EVs, the intensity and duration of these stimuli should be quantified and analyzed to ensure that they do not cause any safety problems. In addition, it should be noted that effective concentrations of certain molecules only work for specific cell lines. Therefore, efforts need to be made to optimize the dose of molecules for each cell line.

#### 4.1.4 Manipulate the MSC gene to enrich the main effector in EV cargo

Targeted modification of MSC-EV through engineering techniques can better improve the efficiency of MSC-EV as well as compensate for the relative lack of EV production (Man et al., 2020). Modification of the parental cell genome allows the EV to be specifically enriched with desired cargo. These cargo may play important therapeutic roles (Hu et al., 2021).

Clarifying the effector components of MSC-EV is critical to elucidate its mechanism of action. According to existing studies, numerous miRNAs and proteins are effector molecules of EV in the treatment of liver disease (Table 1). Using genetic modification to increase the amount of these effectors in MSC-EV can improve the anti-inflammatory, anti-fibrotic, and anti-apoptosis ability of MSC-EV, thereby improving the hepatoprotective effects. For example, miR-122 modification enhanced the efficacy of MSC and exosome for the treatment of carbon tetrachloride (CCl4)-induced liver necrosis by inhibiting HSC activation and attenuating collagen deposition (Lou et al., 2017). Currently, most of the research related to liver diseases only focuses on using plasmids or viral vectors to transfect MSCs. However, gene modification methods such as electroporation and lipid transfection can also increase the expression of their therapeutic effectors. Apart from enhancing the efficacy of EVs, gene modification methods may also realize an increase in the production of EVs. For example, the MSC line overexpressing HIF1-α and telomerase can simultaneously enhance the immunosuppressive efficacy and scale up the production of MSC-EV (Gómez-Ferrer et al., 2020).

Genetic modification also promises to replace the effect of exogenous stimulation. Systematic comparing of downstream mechanisms of the wide range of physical, biological, and chemical stimulants, may lead to the discovery of “common switches” within the MSC. Subsequently, genetic engineering can be used to directly turn on the “switch” of MSC in response to external stimuli, to replace the exogenous stimuli to enhance the amount and efficiency of EV secretion.

### 4.2 Improved rotation rate of the MSC-EV in the liver

Apart from improving the efficacy and yield of MSC-EV, scientists are also working to optimize the *in vivo* processes of MSC-EV. Efforts are first to be made to improve the hepatic targeting of MSC-EV, which could help to enhanced its efficacy in delivering therapeutic bioactive to the damaged liver, as well as to reduce the accumulation of MSC-EV in non-targeted organ and reduce potential side effects and toxicity, thereby improving the therapeutic applicability and safety of MSC-EV therapies. In addition, rapid clearance by the circulating immune system is also one of the bottlenecks limiting the clinical application of MSC-EV. Therefore, prolonging the half-life of MSC-EV is also crucial for improving the rotation rate of MSC-EV. Current strategies can be largely grouped into three main categories: approaches that focus on (Hu et al., 2023) modifying the surface molecules of MSC-EV to improve its liver targeting and evade capture by the immune system (Yang et al., 2021), encapsulating MSC-EV in biomaterials to improve the half-life of MSC-EV, and (Bruno et al., 2021) selecting appropriate delivery strategies to reduce the *in vivo* pathway of MSC-EV in non-target tissues.

#### 4.2.1 Building targeted delivery system

Systemic injection showed that unmodified exosomes were mainly taken up by the mononuclear phagocyte system in circulation. Designing delivery systems with specific affinities can improve the hepatic targeting of MSC-EV, which can mainly be achieved by modifying the surface membrane properties of the EV (Salunkhe et al., 2020). The engineering modification of MSC-EV can be divided into indirect modification and direct modification. Indirect modification refers to the expression of functional peptides or proteins on the membrane of parental cells through genetic engineering, then indirectly obtaining exosomes with membrane protein characteristics similar to those of parental cells; direct modification refers to direct modification of the isolated exosomes, such as manipulate the functional groups on the surface of the EV through chemical coupling reactions, ligand-receptor interactions, multivalent electrostatic interactions, lipid fusion (Mentkowski et al., 2018; Villata et al., 2020).

Membrane components on the EV lipid bilayer (including proteins, lipids, and glycans) (Harrell et al., 2019) and surface properties (e.g., surface charge, lipophilicity) enabling homing, adhesion, uptake and trafficking effects of EV (Shimoda et al., 2017; Edelmann and Kima, 2022). The transmembrane proteins such as Lamp, GPI, and tetraspanins like CD63, CD9, and CD81 on the EV membrane can be fused with targeting ligands for enhancing site specific delivery of exosomes (Kooijmans et al., 2016a; Salunkhe et al., 2020). Genetic engineering of exosome-producing cells using plasmid vectors (encoding targeting ligand fused with one of the above-mentioned transmembrane proteins) is widely used for producing surface modified EVs.

Lamp2b protein is the most used site to fuse with targeting moieties for adhesion purpose (Salunkhe et al., 2020). HSTP1 can be fused with Lamp2b and be displayed on the exosome surface (HSTP1-Exos) through genetic engineering technology. *In vivo* studies have shown that HSTP1-Exos could specifically target the HSC region after intravenous injection and enhance the therapeutic efficacy against hepatic fibrosis (Lin et al., 2022). In addition, Michelle E. Hung et al. found that conjugating glycosylation sequence (GNSTM) with lamp2b could prevent the degradation of lamp2b protein-linked targeting peptides and also increasing the overall expression of Lamp2b fusion proteins in both cells and exosomes, thereby enhancing the efficacy of exosome-linked targeting peptides (Hung and Leonard, 2015). Pullulan is a biomaterial with a high affinity for the liver. It has been found that modification of cationized pullulan onto the surface of exosomes by multivalent electrostatic interaction can increase the hepatic targeting of MSC-Exo. In a mouse model of Con-A-induced acute liver injury, the accumulation of intravenously injected pullulan-modified exosomes in the liver was increased compared to unmodified exosome (Tamura et al., 2017). In addition to pullulan, PEG can also be chemically modified on the surface of exosomes to increase target cell uptake by reducing anion-anion electrostatic repulsion between the exosome surface and the cell surface (Kooijmans et al., 2016b). Some researchers have also modified near-infrared fluorophores (NIR) on exosome surface proteins and found that anionic exosomes showed high hepatobiliary uptake (Hwang et al., 2019). It has also been found that modifying arginine-rich cell-penetrating peptide (CPP) on the surface of EV can induce active macropinocytosis and increase EV uptake by cells (Nakase et al., 2017). STAT3 is a signaling molecule highly expressed in the liver. It has been shown that MSC-Exo loaded with siRNA (iExo^siRNA−STAT3^) or ASO (iExo^mASO−STAT3^) targeting STAT3 via electroporation enables hepatic stellate cell HSC targeting. iExo^siRNA−STAT3^ or iExo^mASO−STAT3^ can better inhibit ECM deposition in liver fibrosis of mice and significantly improve liver function (Tang et al., 2021).

All of the above methods are expected to provide implications for hepatic targeting and hepatic uptake of EV. However, the degradation of the peptide by endosomal proteases in the cell during exosome formation makes it challenging to have the desired yield of peptide-functionalized exosomes (Salunkhe et al., 2020).

Currently, the use of Artificial Intelligence (AI) to design organ-targeting peptides on the surface of MSC-EV has excellent potential. AI can be used to mine large amounts of bioinformatics data, including genomic, proteomic, and transcriptomic data. High-throughput screening techniques, molecular docking, and simulation techniques, as well as machine learning and model prediction, are used to screen, identify, predict, and characterize peptide sequences, protein expression patterns, and signaling pathways with organ-targeting properties, thus identifying potential targeting peptides (Lin et al., 2022; Mahajan et al., 2022). However, it should be noted that although AI can help screen peptide sequences with potential targeting properties, the final synthesizability requires further experimental validation and optimization.

In conclusion, constructing a targeted delivery system provides a more precise, effective, and safe method for treating MSC-EV, which is expected to further enhance its therapeutic efficacy.

#### 4.2.2 Evading immune elimination

Recognition and clearance by the immune system have a significant impact on the *in vivo* metabolism and distribution of MSC-EV in specific tissues. It has been shown that the recognition of MSC-EV by immune cells is mainly mediated through the membrane proteins and surface properties of EV. Therefore, modification of the membrane proteins and surface properties of EV could help MSC-EV to evade immune phagocytosis, thus prolonging the half-life *in vivo*.

Several receptors with escape immunorecognition capacity are present on the surface of EVs. By binding or overexpressing specific antibodies, it is possible to reduce their interaction with immune cells and prolong their half-life in the body. Particularly, CD47 expressed on the membrane of MSC-EV, providing a caution to macrophages via CD47-SIRPα signaling, which can help MSC-EV escape from the clearance and uptake of circulating monocytes (Kamerkar et al., 2017). Overexpression of CD47 on the surface of EV increased its *in vivo* half-life to 3-fold (Yang et al., 2020).

The polarity of the MSC-EV surface is also an important factor influencing its targeting properties and recognition by immune cells. Anisotropic membrane charge can promote EV-cell interactions, such as affecting the preferential uptake of MSC-EV with hepatocytes (positive charge) and kupffer cells (negative charge) (Driscoll et al., 2021). Positively charged Particles (NPs) were found to accumulate mostly in the liver, whereas neutral and negatively charged NPs tended to be uptake by the mononuclear phagocyte system (MPS) (Blanco et al., 2015). Phosphatidylserine (PS) on the surface of exosomes is enriched with the negative charge, and macrophages may recognize EV through the negative charge of PS, resulting in exosomes being cleared by the immune system (Matsumoto et al., 2017). Modifying the surface of MSC-Exo with PEG can convert the negative surface charge to a positive charge, which can help exosomes escape the lysosome-mediated endocytosis pathway and reduce the clearance rate of MSC-EV (Tamura et al., 2017). This suggests that the immune response to EV *in vivo* is influenced by its surface composition. However, the relationship between differences and variations in EV surface composition and the rate of hepatocyte uptake and recognition of MSC-Exo by immune cells needs further investigation.

Besides, strategies such as selecting MSC-EV subpopulations with higher immune escape capabilities and employing immunosuppressive agents can also effectively mitigate the immune system’s recognition and clearance of MSC-EV, but the exact method of implementation needs to be further researched.

#### 4.2.3 Loading EV into biomaterials

Sustained-release MSC-EV has been proposed as a new strategy to prolong the bioavailability in the target liver. By changing the composition or envelope structure of MSC-EV, the release rate of MSC-EV can be controlled to maintain the plasma concentration of MSC-EV at a relatively stable level. Optimization of MSC-EV formulation technology and preparation of sustained and controlled release delivery systems can prolong the half-life of MSC-EV *in vivo*. The ideal delivery technology for delivering MSC-EV should have the following characteristics (Hu et al., 2023): good biocompatibility (Yang et al., 2021); the ability to target or stay in the specific tissue (Bruno et al., 2021); sustained-release function, which means the MSC-EV can be released for a long period time after encapsulation in a stable and sustained manner.

Among various biomaterials developed for EV delivery, hydrogel is the most promising. Hydrogel is a three-dimensional network gel formed by hydrophilic polymers through physical or chemical cross-linking (Murali and Holmes, 2021). The rate of exosome release depends largely on the pore size and cross-linking density of the hydrogel, and the swelling and degradation process of the hydrogel allows for sustained exosome release *in vivo* (Huang et al., 2021). The material properties of the hydrogel, such as porosity and degradability, can be designed based on the molecular particle size and half-life of the MSC-EV in advance, enabling the EV to be released continuously and stably at a suitable rate (Wechsler et al., 2021; Ju et al., 2023). The functional hydrogel can even be triggered to release EV by specific stimuli in the microenvironment *in vivo* (enzymes, light, temperature, pH, and other stimuli), thus extending the half-life of EV *in vivo* (Murali and Holmes, 2021). In addition, hydrogel is highly biocompatible and easy to modify (Pinheiro et al., 2018; Lu et al., 2022). Currently, the strategy of using hydrogel as a carrier for MSC-EV has been widely used in bone (Zhou et al., 2021), cartilage (Watanabe et al., 2021), kidney (Zhou et al., 2019), heart (Hazrati et al., 2022), and nerve (Wu and Meng, 2021).

Polyethylene glycol (PEG) is widely used for nanoparticles to escape MPS removal, greatly extending the circulation time of nanoparticles in the body (Wen et al., 2023). The researchers mixed clickable polyethylene glycol (PEG) macromeres with MSC-EV to form EV-encapsulated PEG hydrogels (Gel-EV) via a fast, biocompatible click reaction. After injecting Gel-EV into mice with chronic liver failure, Gel-EV could be continuously released within 4 weeks (through the gradual biodegradation and swelling properties of the hydrogels), which prolonged the half-life of EV and increased the hepatic accumulation. The bioavailability of MSC-EV was improved by nearly 50%, while free-EV was removed from the blood and liver within 24 h (Mardpour et al., 2019).

Another study encapsulated the MSC secretome in a PLG and further encased this particle in RBC membranes to make MRIN products. Studies have shown that this MRIN product evaded macrophage recognition and increased MSC secretome’s stability and retention time *in vivo*. Intravenous injection of MRIN improved the survival rate of mice in acute liver failure. In addition, MRIN supports long-term frozen storage after lyophilization, making it easier to prepare and preserve (Liang et al., 2018).

However, although biomaterials such as hydrogel have been widely used in MSC-EV delivery, there are still unresolved issues in the clinical translation of the delivery technology. For example, the binding of EV to the delivery material can be affected by donor heterogeneity, resulting in inconsistent release profiles (Murali and Holmes, 2021). In addition, there are no reports on the storage and effectiveness of EV-loaded hydrogels (Ju et al., 2023). The research on MSC-EV delivery technology in liver diseases is still incomplete. The therapeutic potential in liver diseases of technologies such as implantable scaffolds and biomaterial membranes (Pinheiro et al., 2018), which are widely used in other systemic diseases, needs further investigation.

#### 4.2.4 Select the appropriate administration route

The administration route determines the route and speed of entry of MSC-EV into the body, thus affecting its distribution in different tissues and organs. The appropriate administration route should minimize the pathway of EV getting into the liver and reduce its residence time in circulation. In addition, the administration should be as convenient, simple, and non-invasive as possible to minimize patient burden (Varderidou-Minasian and Lorenowicz, 2020). Based on the existing preclinical studies, the routes of administration of MSC-EV can be mainly categorized into systemic delivery and *in situ* delivery.

Current local hepatic administration of MSC-EV includes hepatic portal vein injections (Xie et al., 2019a), intrasplenic injection (Tan et al., 2014; Qu et al., 2017; Mardpour et al., 2018), Intrahepatic injection (Li et al., 2013). These administration routes have now been applied in models of hepatic IRI, liver injury, cirrhosis and other liver diseases. In comparison to systemic delivery, hepatic *in situ* delivery reduces *in vivo* transport pathways, circulating immune system clearance, and the enrichment of non-target organs of MSC-EV, which implies that MSC-EV can achieve the desired therapeutic effect in target tissues with lower doses. However, due to the complexity of the trauma environment, localized delivery of EVs is often susceptible to degradation and failure upon direct entry into the inflammatory or injurious environment (Li and Wu, 2022). Therefore, whether *in situ* administration can definitively improve the *in vivo* utilization and efficacy of MSC-EV needs to be further explored. In addition, since *in situ* administration is more invasive, the route of administration should be considered in terms of patient tolerance when used in the clinical setting.

Currently, systemic drug delivery is still the most widely used mode of drug delivery in preclinical studies due to its low invasiveness and convenience. It includes intravenous administration, arterial administration, oral administration and intraperitoneal administration. Intravenous injection (IV), where no absorption process exists, is the most common mode of administration for MSCs and MSC-EV. MSC-EV can flow throughout the body and accumulate in the liver rapidly by directly injecting into the blood circulation (Pinheiro et al., 2018; Royo et al., 2019). However, due to the non-targeted diffusion and presence of macrophages in circulation, intravenous injection may result in rapid clearance of MSC-EV (Imai et al., 2015). Thus the short half-life index is one of the main limitations of IV administration (Takahashi et al., 2013). Oral administration is less invasive than intravenous administration. However, MSC-EV needs to overcome changes in gastrointestinal pH, enzyme activity, digestion by intestinal flora, and penetration of the intestinal mucosal barrier (Pinheiro et al., 2018). When oral gavage and intravenous injection MSC-EV were used to treat ALF mice in acute liver failure, the oral administration group detected fewer EVs *in vivo* and a lower overall survival rate of mice compared to the IV group. In preclinical studies, the intraperitoneal injection (IP) of MSC-EV is also a viable option for systemic drug delivery, which can accommodate a higher upper dose limit (Pinheiro et al., 2018; Rezaie et al., 2018). Nevertheless, intraperitoneal administration may lead to the dilution of MSC-EV and off-target diffusion (Haga et al., 2017). In addition, the clinical feasibility of intraperitoneal administration also requires further study.

Since most of the studies were preclinical animal studies, there is no conclusive evidence as to which dosing regimen is best for the treatment of liver disease with MSC-EV. When considering the clinical dosing regimen of MSC-EV, the administration regimen should be designed based on the indication, target organ, timing of administration, and patient tolerance.

## 5 Limitations of MSC-EV in clinical transition

A large number of preclinical studies have demonstrated the efficacy of MSC-EV, providing strong evidence for MSC-EV to become a new therapeutic strategy in liver disease. However, MSC-EV still needs to overcome some bottlenecks (Hu et al., 2023): establishing standards for production and quality control processes (Yang et al., 2021); building the clinical efficacy and adverse reaction evaluation system.

For producing standardized MSC-EV products, it is first necessary to identify the source of MSC donor cells. The character of variability in growth, differentiation potential, and immunomodulatory potential of MSC isolated from different donors needs to be taken into account, as the MSC donor’s age (Charif et al., 2017; Yin et al., 2017; Fafián-Labora et al., 2019; Adlerz et al., 2020), gender (Katsara et al., 2011), BMI (Oñate et al., 2012; Ulum et al., 2018), and health status (Costa et al., 2021) all influence the quality of the MSC. When selecting donors of MSC, the screening criteria should be standardized as much as possible, and detailed screening and recording of the donor’s physical condition, age, smoking, infectious diseases, family history of hereditary diseases, and relevant medical records should be carried out. In addition to the physiological condition of the donor of MSCs, the tissue source also affects the properties of MSCs. Currently, MSC-EV derived from bone marrow (Rong et al., 2019), adipose (Jin et al., 2018), umbilical cord (Jiang et al., 2018), embryo (Mardpour et al., 2018), amnion (Ohara et al., 2018), tonsil (Kim et al., 2021), placenta (Jun et al., 2020), iPSC(Du et al., 2017a) and liver (Wang et al., 2016; Bruno et al., 2020; Bruno et al., 2021) have all demonstrated hepatoprotective effects in preclinical studies (Table 1). However, only MSCs isolated from adipose tissue, bone marrow or umbilical cord have been used in clinical trials (Lotfy et al., 2023). Although MSC-EV products from different tissue sources have basic biological functions, they still differ in MSC self-replication rates (Zhang et al., 2011), EV-secreting amounts (Ragni et al., 2017), and EV efficacies (Lopez-Verrilli et al., 2016). Such as in the rat model of TAA-induced chronic liver fibrosis, MSC derived from human embryonic can significantly suppress the proliferation of peripheral blood mononuclear cells compared to MSC derived from bone marrow and adipose (Mardpour et al., 2018). Identifying the most promising subpopulations of tissue-regenerating MSC-EV may be valuable for maximizing the therapeutic outcome of liver disease. In addition, the EV secretory activity and bioactivity of MSC decreased significantly after several cell passages, and this decrease may be related to the senescence of MSC (Patel et al., 2018). For obtaining MSC-EV with high therapeutic efficacy, it may be a reasonable choice to select the EV produced by the 4th–7th generation MSCs (Willis et al., 2017). However, it has also been shown that the angiogenic vascularization bioactivity of MSC-EV is significantly reduced when MSC is digested by trypsin beyond the 4th generation (Patel et al., 2017). The relationship between the specific passage number and biological activity of MSC may vary depending on the experimental conditions and design of the study. The optimal MSC passages for harvesting EV are currently inconclusive.

After determining the cell source of EVs, optimized MSC culture parameters can also improve the replication rate and cell viability of MSC, as well as MSC-EV production (Phan et al., 2018). These culture parameters include selecting the appropriate media, cell implantation density, culturing time, and frequency of EV collection. Serum-free culture is a culture method that does not use animal serum as a nutrient but adds growth factors to avoid potential pathogen contamination from animal serum (Yin et al., 2019). Currently, serum-free media have been widely used in the cultivation and production of MSC-EV. It has been shown to enhance the capacity of MSC-EV (Bobis-Wozowicz et al., 2017; Kim et al., 2021). Except for selecting the appropriate medium substrate, determining the MSC seeding density and frequency of EV collection also affects the yield and function of MSC-EV. It has been reported that reducing the cell seeding density in culture flasks can increase the EV yield of individual MSCs, and more frequency of EV collection can increase the total yield (Patel et al., 2017).

In addition, it is important to ensure the quality of MSC-EV products and reduce lot-to-lot variation. There is also a need to assess the quality of MSC after production. The lack of standardization in the production of MSC-EV has led to batch-to-batch heterogeneity between the same and different laboratories (Zhou et al., 2021). It is essential to ensure consistent product quality from generation to generation, including sterility, safety, purity, activity, identity, and stability. However currently used to assess the characteristic of MSC-EV metrics mainly including the numbers, concentration, size, morphology, surface markers (such as CD9, CD81 and CD63), while for the biological activity of MSC-EV and related detection indicators have no unified standard or common method. Currently, the functions of MSC-EV can be evaluated in experimental models, such as tissue repair, anti-inflammatory and immunomodulatory abilities (Li et al., 2018). In addition, MSC-EV needs to develop biological indicators closely related to its indications, such as liver enzyme and liver structure damage indicators. In summary, the development of new methods and techniques to systematically evaluate the biological activity and efficacy of MSC-EV products is of great significance for establishing quality control guidelines for MSC-EV and advancing its clinical application.

Finally, the safety of cell-free products in clinical use (including toxicity, immunogenicity, and potential side effects), requires a thorough assessment and contingency planning. Theoretically, MSC-EV express relatively few molecules that can induce immune responses, and do not have the ability of multi-directional differentiation and self-replication, which reduces the risk of their immunogenicity and proliferation into tumors *in vivo*, making them relatively safer in treatment (Murphy et al., 2019). But although MSC-EV are widely accepted in most cases, differences between individuals (including immune status, medical history, physiological health) may affect the response of patients to MSC-EV. At present, some clinical trials have explored the safety of MSC-EV (NCT04491240 and NCT05523011), and no adverse reactions have been found in patients with Psoriasis and COVID-19 when used MSC-EV (Sengupta et al., 2020). Presently, there are already 7 completed clinical studies and 14 ongoing clinical studies on MSC-EV, including osteoarthritis, stroke, Alzheimer’s disease, type 1 diabetes and other diseases, but there are no completed studies in liver disease at this point of time (Lotfy et al., 2023). In addition, there are few studies on the minimal effective doses (MEDs), minimal toxic doses, and safety range of MSC-EV in liver diseases. Whether MSC-EV will cause adverse reactions when used in clinical liver diseases is still unknown. More research and evaluation are needed before applying it to clinical treatment to ensure the safety and efficacy of MSC-EV.

## 6 Conclusion

A growing number of studies have shown the therapeutic efficacy of MSC-based cell-free products, which makes MSC-EV a promising alternative to MSC for treating liver diseases (Table 1). However, current studies have yet to resolve the yield, efficacy, and rotation rate problems of MSC-EV for clinical application. Therefore, we summarized engineering strategies that offer potential ways to further optimize the therapeutic efficacy and stability of MSC-EV in treating liver diseases (Figure 1; Table 2), to promote the development and clinical transition of MSC-EV. As described above, first we can modify the parental cells of EV by indirect methods. Strategies such as hypoxia induction, 3D culture, exogenous stimuli and gene regulation were used to increase the yield and the therapeutic factors secretion in the cargo of MSC-EV, thus enhance the therapeutic activity. In addition, we can directly modify EVs, including adjusting the surface properties of MSC-EV and optimizing the route of administration for liver-targeting and immune evasion effects, thus improving the conversion rate of EVs *in vivo*. Taking advantage of these bioengineering techniques allows cellular processes and *in vivo* fate to be adapted to EV functionalization.

**TABLE 2:** Example of improving the therapeutic efficacy of MSC-EV in liver disease.

Strategy category	Method	Cell source	Type of EV	Liver disease	Advantages	Ref.
hypoxia induction (Improve the therapeutic efficacy)	culture MSCs under hypoxia (10%, 5% and 1% pO_2_) for 24 h	adipose MSC	CM	liver transplantation/liver regeneration	• Reduced the inflammatory factors (TNF-α and IL-6) and function-associated indicators (ALT and AST)	Lee et al. (2017)
					• Upregulated the expression of hepatocyte proliferation and anti-apoptotic markers	
					• Inhibited the expression of pro-apoptotic markers	
	culture MSCs under hypoxia (10% O_2_)	mouse bone marrow MSC	exosome	acute liver failure (ALF)	• significantly improved the biochemical parameters	Temnov et al. (2019a)
					• reduced the degree of inflammation	
					• stimulated regenerative processes in liver	
	culture MSCs under 5% CO_2_, 94% N_2_ and 1% O_2_ for 24 h in a hypoxic chamber	mouse bone marrow MSC	exosome	acute liver injury (ALI)	• Significantly enriched miR-182-5p	Xu et al. (2022)
					• Induced anti-inflammatory response by inhibiting the expression of FOXO1 protein in macrophages and TLR4 expression	
					• Enhanced M2 macrophage polarization both *in vivo* and *in vitro*	
3D culture (Improve the therapeutic efficacy)	hanging drop culture for MSC to formed 3D spheroids	human adipose MSC	CM	liver fibrosis	• 3D spheroids of MSCs exhibited higher levels of IGF-1, IL-6, and HGF compared with normally cultured MSCs	Zhang et al. (2016b)
					• enhanced the expression of antifibrotic factors by MSCs, including IGF-1, IL-6 and HGF	
					• protected hepatocytes from cell injury and apoptosis more effectively	
					• Ameliorated hepatic fibrosis and improved liver function	
Exogenous stimulation (Improve the therapeutic efficacy)	pretreate MSC with 1 ng/mL TNF-α for 3days	human umbilical cords MSC	exosome	acute liver injury (ALI)	• significantly decreased the secretion of inflammatory factors (IL-1β, IL-18 and IL-6) in Kupffer cells, hepatic injury and function-associated indicators (ALT and AST)	Zhang et al. (2020a)
					• inhibited activation of NLRP3 inflammation-associated pathway protein	
	Stimulate MSC with 1 ng/mL IL-6 for 48 h	human umbilical cords MSC	exosome	acute liver injury (ALI)	• Enriched miR-455-3p in exosomes derived from MSC pretreated with IL-6	Shao et al. (2020)
					• inhibited the activation of macrophages both *in vivo* and *in vitro* by potentially targets PI3K signaling	
					• attenuate local liver damage and reduce the serum levels of inflammatory factors (IL-6, G-CSF, IL-17, IL-10, IP-10 and MCP-1)	
	pretreated MSC with 100 ng/mL human recombinant IFN-γ for 48 h	human adipose MSC	EV	liver cirrhosis	• EV revealed anti-inflammatory macrophage inducible proteins (e.g., annexin-A1, lactotransferrin, and aminopeptidase N)	Takeuch et al. (2021)
					• ameliorated inflammation and fibrosis in the cirrhosis mouse model more effectively	
					• increases the abundance of anti-inflammatory macrophages and induces multipotent effects by increasing the abundance of Treg cells	
	pretreated MSC with H2S donor (NaHS at 1 μmol) in an FBS-free medium.	human umbilical cords MSC	exosome	hepatic ischemia-reperfusion injury (HIRI)	• enhance the therapeutic effects of MSC-derived exosomes	Sameri et al. (2022)
					• improved liver function by reducing inflammatory cytokines (TNF-α and IL-6), cellular apoptosis, liver levels of total oxidant status, and liver aminotransferases (ALT and AST)	
	pretreated MSC with Baicalin	bone marrow MSC	exosome	acute liver injury (ALI)	• significantly upregulated P62 in Exosome derived from MSC pretreated with Baicalin	Zhao et al. (2022)
					• Attenuated LPS/D-gal-induced liver damage and inhibits reactive oxygen species (ROS) production	
					• regulated hepatocyte ferroptosis by activating the Keap1-NRF2 pathway	
	Coculture MSCs with hepatocytes	bone marrow MSC	CM	acute liver failure (ALF)	• Prolonged the survival time of ALF rats	Chen et al. (2018b)
					• Prevented liver injury and promoted liver tissue repair	
	induced MSCs to differentiate into hepatocyte‐like cells and extracted their exosomes	bone marrow MSC	exosome	hepatic ischemia-reperfusion injury (HIRI)	• increased hepatocyte tolerance to ischemia and reduced hepatocyte apoptosis	Yang et al. (2020a)
					• enhanced autophagy	
Genetic-manipulation (Improve the therapeutic efficacy)	Overexpress miR-223: infect MSC with Lentivirus encoding miR-223	mouse bone marrow MSC	exosome	autoimmune hepatitis (AIH)	• Reversed either S100 or LPS/ATP induced injury in mice and hepatocytes	Chen et al. (2018a)
					• Downregulated the release of NLRP3 and caspase-1	
	Overexpress miR-223-3p: infect MSC with Lentivirus encoding miR-223-3p	mouse bone marrow MSC	exosome	autoimmune hepatitis (AIH)	• Attenuated inflammatory responses in both the liver and macrophages	Lu et al. (2019)
					• downregulated STAT3 gene and inflammatory cytokines (IL-1β and IL-6) expression	
					• reduced ratio of Treg/Th17	
	Overexpress miR-181-5p: infect MSC with Plasmids encoding miR-223-3p	mouse adipose MSC	exosome	liver fibrosis	• attenuated liver injury	Qu et al. (2017)
					• significantly downregulated collagen I, vimentin, a-SMA and fibronectin in liver	
					• downregulated STAT3 and Bcl-2 and activated autophagy in the HST-T6 cells	
					• activated autophagy by upregulating Beclin1 expression	
					• inhibited the expression of proinflammatory factors TNFa, IL-6, and IL-17	
	Overexpress MiR‐122: infect MSC with lentivirus encoding miR-223-3p	mouse adipose MSC	exosome	liver fibrosis	• suppressed the activation, proliferation of HSCs	Lou et al. (2017)
					• alleviated collagen deposition	
	Overexpress circDIDO1: Full-length circDIDO1 was cloned into the pLC5-ciR vector, and the plasmid was transiently transfected into MSC	human bone marrow MSC	exosome	human liver fibrosis	• suppressed HSCs activation by elevating PTEN to suppress AKT pathway through sponging miR-143-3p	Ma et al. (2022a)
	Overexpress CDK13: infect MSC with plasmid encoding CDK13	human bone marrow MSC	exosome	liver fibrosis	• reduced HSC cell activity and decreased the expressions of vimentin, desmin, α-SMA, collagen I, fibronectin and MMP9	Ma et al. (2022b)
					• inhibited PI3K/AKT and NF-κB signaling pathways activation through regulating the miR-17-5p/KAT2B axis	
	Overexpress miR-150-5p: infect MSC with lentiviral encoding miR-150-5p	mouse adipose MSC	EV	liver fibrosis	• inhibited the CXCL1 expression	Du et al. (2021)
					• inhibited CVF (indicator of liver injury), reduced the levels of inflammatory factors (TNF-α, IL-6 and IL-17), hepatic injury and function-associated indicators (ALT, AST and TB)	
					• decreased The expression levels of CXCL5, collagen I, collagen III and fibronectin	
	Overexpress miR‐20a	human umbilical cords MSC	exosome	liver ischemia/reperfusion injury	• almost fully alleviated I/R injury	Zhang et al. (2020b)
					• improved the therapeutic effect by inhibiting Beclin1‐ and FAS‐mediated autophagy andapoptosis	
Surface modification (↑targeting)	The siRNA and ASO were designed to target mouse and human STAT3. Load siRNA or ASO in exosome by electroporation.	bone marrow MSC	exosome	liver fibrosis	• enhanced STAT3 targeting efficacy	Tang et al. (2021)
					• suppressed STAT3 levels and ECM deposition in established liver fibrosis in mice	
					• significantly reduced the percentage of hepatocytes with necrosis and degeneration	
					• improved liver function (iExo^mASO−Stat3^ restored liver function more efficiently when compared to iExo^siRNA−STAT3^)	
	Mix Collected exosome with the cationized pullulan, and through an electrostatic interaction of both substances	MSC	exosome	liver injury	• promoted the uptake of MSC-EV *in vitro*	Tamura et al. (2017)
					• increased accumulation in the liver tissue, resulting in an enhanced anti-inflammatory effect *in vivo*	
	screened the HSTP1 peptide from the phage display peptide library. Then, fused HSTP1 with exosomal enriched membrane protein (Lamp2b) and displayed them on the surface of exosomes through genetic engineering technology.	human umbilical cords MSC	exosome	liver fibrosis	• HSTP1 can specifically bind to HSCs and as a promising molecular imaging probe for the pathological diagnosis	Lin et al. (2022c)
					• realized the precise treatment of nanomedicine for a single type of cell in complex liver tissue	
					• improved the ability of exosomes to reverse liver fibrosis: reduced Collagen deposition and; regulate M2 macrophage polarization by inhibiting CCl2 secretion from aHSCs	
Encapsulation (↓immune system recognization)	encapsulate EVs with PEG hydrogels via biocompatible click reaction	human embryonic stem cell (ES-MSC)	EV	chronicliver fibrosis	• the accumulationof EVs in the liver was extended by hydrogel-mediated delivery for 1 month. Four weeks after injection in a rat model, the harvested liver showed superior antifibrosis, anti-apoptosis, and regenerative effects of the EVs	Mardpour et al. (2019)
					• improved the anti-fibrosis, anti-inflammation, anti-apoptosis, and regenerative effects of the EVs to nearly 40, 50, 40, and 50%	
	coated MSC-CM with the PLGA particles and the membranes of red blood cells	human bone marrow MSC	CM	acute liver failure (ALF)	• protected the MSC-CM from recognition by macrophages and increased the blood stability of MSC-CM	Liang et al. (2018)
					• had great liver retention after intravenous delivery	
					• reduced the levels of inflammatory factors (TNF-α, IL-1βand IL-6), hepatic injury and function-associated indicators (ALT and AST)	
					• support long-term cryostorage after lyophilization	

In the age of precision medicine, we speculate that future MSC-EV products may evolve in two directions: The first is to make the potency of MSC-EV more customized, personalized and Fit-for-Purpose. For the treatment of liver disease, this may involve adjusting the cargo composition, release rate, frequency of administration, and organ-specific targeting of the MSC-EV to ensure it is highly tailored to the patient’s tolerance, indication, state of an illness, or even genotype. Another development direction is to produce generic MSC-EV. This means that the manufacturing process will follow strict GMP management and monitoring standards to ensure the quality and consistency of each batch of MSC-EV products, which allows for greater reproducibility of MSC-EV products to meet broad or urgent clinical needs. Such generic and ready-to-use MSC-EV products can be more easily applied to different clinical scenarios, reducing the complexity of production and distribution. These two development directions are not mutually exclusive but can complement each other. Depending on different clinical needs and market demands, patients can choose to adopt customized MSC-EV products or generic MSC-EV products to reach optimal therapeutic effects and convenience. It will help promote the further development and application of MSC-EV technology.
